# Feeding Rhythms and the Circadian Regulation of Metabolism

**DOI:** 10.3389/fnut.2020.00039

**Published:** 2020-04-17

**Authors:** Lauren Pickel, Hoon-Ki Sung

**Affiliations:** ^1^Translational Medicine Program, The Hospital for Sick Children, Toronto, ON, Canada; ^2^Department of Laboratory Medicine and Pathobiology, University of Toronto, Toronto, ON, Canada

**Keywords:** circadian, metabolism, time-restricted feeding, fasting, high fat diet, ketogenic diet, peripheral clock

## Abstract

The molecular circadian clock regulates metabolic processes within the cell, and the alignment of these clocks between tissues is essential for the maintenance of metabolic homeostasis. The possibility of misalignment arises from the differential responsiveness of tissues to the environmental cues that synchronize the clock (zeitgebers). Although light is the dominant environmental cue for the master clock of the suprachiasmatic nucleus, many other tissues are sensitive to feeding and fasting. When rhythms of feeding behavior are altered, for example by shift work or the constant availability of highly palatable foods, strong feedback is sent to the peripheral molecular clocks. Varying degrees of phase shift can cause the systemic misalignment of metabolic processes. Moreover, when there is a misalignment between the endogenous rhythms in physiology and environmental inputs, such as feeding during the inactive phase, the body's ability to maintain homeostasis is impaired. The loss of phase coordination between the organism and environment, as well as internal misalignment between tissues, can produce cardiometabolic disease as a consequence. The aim of this review is to synthesize the work on the mechanisms and metabolic effects of circadian misalignment. The timing of food intake is highlighted as a powerful environmental cue with the potential to destroy or restore the synchrony of circadian rhythms in metabolism.

## Introduction

The rotation of the Earth produces daily and seasonal changes in the environment to which organisms have become adapted. The self-sustaining oscillations in physiology produced in approximation of the 24 h daily cycle that evolved to exploit these changes are referred to as circadian rhythms (from the Latin *circa* and *diem*, about a day). The anticipation of reliable patterns in the environment evolved through over two trillion day–night cycles (1). Circadian biology is pervasive from protozoans, cyanobacteria and algae to plants, fungi, and animals (2), and the molecular mechanisms that regulate it are similar among the kingdoms of life, indicating more than 500 million years of positive selection (3). The present review focuses on the mammalian system. The selective pressures under which the circadian rhythms evolved were fundamentally linked to the period of specific environmental oscillations, such as light/dark, which became cues for the *entrainment* of the endogenous clock period. It is the reliable prediction of regular environmental changes that make circadian biology advantageous. Rhythms misaligned to the environment are not only neutral but detrimental to organismal fitness (4). For example, mice with an endogenous period substantially shorter than 24 h (due to the *tau* mutation in casein kinase 1ε) have greatly reduced fitness (5).

Within an organism, circadian rhythmicity is pervasive at all levels of organization, from intracellular molecular networks of transcription and translation to the neuronal networks that produce rhythms at the behavioral level and even the coordination of social activities and reproduction. The most important feature of an endogenous clock is the *phase relationship* between the rhythms it generates and those of the external environment. In peripheral tissues with primarily metabolic functions, the most important phase relationship is between their rhythmic processes and environmentally dictated daily fluctuations in energy input and requirement. Cellular energy demands vary temporally, which requires regulators to orchestrate the oscillations in metabolism (6). Moreover, rhythmic cellular respiration produces metabolites that become damaging if they accumulate, which necessitates synchronized compensation mechanisms, such as reactive oxygen species (ROS) scavengers (7, 8). Opposing biochemical pathways must also be kept separate, and in addition to sequestration in subcellular compartments, this is achieved through temporal separation with inhibitory feedback loops (9). For example, the hepatic clock directly regulates a daily switch between dark phase glycogenesis and light phase glycogenolysis in mice (10).

Most food consumption and physical activity occur during the animal's active phase of the 24-h cycle. For humans and other diurnal species this corresponds to the light phase, and for nocturnal species such as mice, to the dark phase. In both nocturnal and diurnal mammals, the molecular architecture of the circadian system involves the same components, which oscillate in a similar phase with an approximately 24-h period; the opposite phase of activity is the output of homologous circadian physiology. The core molecular clock is also the same across tissues, although the outputs and regulation of the clock are tissue-specific. Food intake is an important synchronizing cue for the clocks of many tissues with essential metabolic roles, and these will be the focus of the present review.

## The Mammalian Molecular Clock

**Figure 1:**
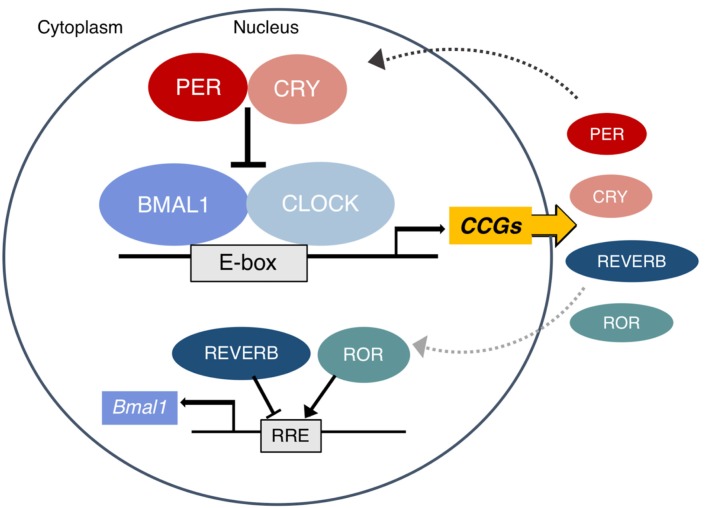
The mammalian molecular clock. The complex of BMAL1 and CLOCK rhythmically binds E-box elements to activate the transcription of clock-controlled genes (CCG), including other parts of the core clock which form the negative arm of the feedback loop. The PER and CRY proteins translated from *Period* (1–3) and *Cryptochrome* (1, 2) translocate to the nucleus and inhibit the activity of CLOCK:BMAL1. Eventually they are tagged for proteasomal degradation and the cycle begins again approximately every 24 h. In a secondary loop, the protein products of the *Rev-erbs* (α and β) and *RORs* (α, β, and γ) inhibit and activate *Bmal1* transcription, respectively. Other CCGs are involved in the pathways of metabolic regulation. A subset of these (including PPARs, PGC1α, AMPK, and NAD+ dependent SIRT1) in turn also regulate the clock. Figure adapted with permission from Gaucher et al. (12).

In mammals, the autonomous oscillator that produces the circadian rhythms observable at all levels of biology is found at the molecular level. The molecular clock is a highly conserved transcription–translation feedback loop in which transcriptional activators drive the production of their own repressors in an ~24 h cycle (11). The core clock feedback loop is described briefly in Figure 1 and was recently reviewed in detail by Takahashi (13).

Epigenetic mechanisms make the metabolic interactions with this core clock highly plastic. Exposing or condensing E-box regions control CLOCK:BMAL1 binding and the ability to regulate entire networks (14). Numerous clock-controlled genes (CCGs) are key metabolic regulators, a topic that has been extensively reviewed (9, 15, 16). Among the CCGs involved in metabolism, a subset directly feeds back into the clock, producing the bidirectional regulation of metabolism and circadian rhythms, which includes all members of the PPAR family [α, β/δ, and γ; (17)], PGC1α, and AMPK (15). The interaction of the clock and nutrient-dependent metabolic regulators is so tight that a distinction between their rhythmic transcriptional outputs is somewhat arbitrary (18). Other direct circadian output genes that act as non-essential modulators of the clock rhythm and have known metabolic functions include the PARbZip transcription factors Dbp, Hlf, Tef, and the repressor Nfil3 (19) as well as Dec1 and Dec2 (20).

## Organization and Entrainment of the Circadian System

The molecular oscillator maintains a phase that closely ~24 h in free-running conditions (without external input), but its *free-running period* does not perfectly match the external day. What allows the circadian system to keep accurate time with the external environment is its plasticity: the clock undergoes daily resynchronization to rhythmically occurring environmental cues called *zeitgebers* (“time givers”). The most reliable daily rhythm in the environment, and therefore a universal zeitgeber across all phylogenetic levels, is the cycle between light and dark.

### The Central Circadian Clock

The mammalian circadian system is classically depicted as having a hierarchical organization with a “master clock” oscillator in the suprachiasmatic nucleus (SCN) of the hypothalamus that synchronizes the phases of all the other molecular clocks in the body (21). Specialized melanopsin-expressing photoreceptor cells in the retina, known as “intrinsically photosensitive retinal ganglion cells,” transduce incoming light and relay this information to the SCN via the retinohypothalamic tract (22). Light causes phase delays in the clock when it is present early in the night, or phase advances when it occurs late at night (23, 24), resulting in effective synchronization of the SCN clock to the external light/dark cycle.

### Synchronization of Peripheral Clocks by the SCN

**Figure 2:**
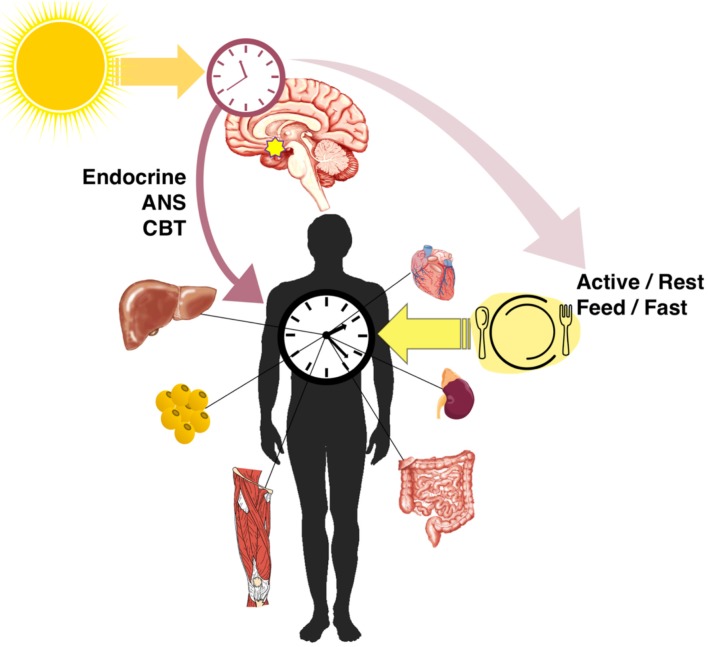
Entrainment and alignment of the SCN and peripheral clocks. The primary zeitgeber of the SCN oscillator is light, whereas peripheral clocks are strongly entrained by feeding/fasting rhythms. The SCN synchronizes peripheral rhythms directly through endocrine and autonomic nervous system (ANS) signals and the regulation of the core body temperature (CBT), as well as indirectly through behavioral feedback from rhythms in activity and feeding.

In addition to the master clock in the SCN, nearly every cell in the body has an autonomous clock (25) which has the components described in Figure 1. The network of pacemaker neurons in the SCN acts as the orchestrator of these self-sustaining oscillators throughout the body, relaying the light-entrained phase by various mechanisms (Figure 2). These include the regulation of core body temperature as well as direct communication through autonomic innervation and endocrine signaling (26), primarily through adrenal glucocorticoids and pineal melatonin (27). As outputs of the master clock, fluctuations in body temperature, plasma cortisol, and melatonin rhythms are used to gauge its phase.

The other important mechanism by which the central and peripheral clocks are aligned is through SCN-generated behavioral rhythms (28), including wake/sleep, activity/rest, and feeding/fasting (Figure 2). Indeed, the SCN is necessary to drive behavioral and feeding rhythms. Early studies of SCN lesions (29) and recent SCN-enriched selective knockouts of clock genes (30, 31) confirmed that without a functional central clock, animals in constant darkness are arrhythmic in their activity and consume similar proportions of food during both light and dark phases. Along with lost activity rhythms, SCN lesioning flattens rhythms in energy expenditure which disturbs energy balance even without differences in total expenditure (32). The SCN-generated rhythms in activity and food intake produce feedback that strongly entrains the clocks of other tissues. When adrenal hormone output (direct SCN control) and food intake rhythms (indirect behavioral control) are eliminated, rhythmic clock gene expression in the liver and white adipose is lost, along with metabolic output genes and adipokines (33, 34).

### Entrainment of Central and Peripheral Clocks

Light and dark cycles are the dominant zeitgeber of the master clock in the SCN, whereas the rhythm of feeding behavior is arguably the most powerful zeitgeber of the peripheral tissues. In a classic study, Damiola et al. (35) showed that restricting food access to the inactive phase in mice (i.e., the light phase) caused a phase shift in the peripheral tissue clocks of the liver, pancreas, heart, skeletal muscle, and kidney. In contrast, the central clock in the SCN was unaffected. This uncoupling between the central and peripheral clocks occurred equally when the animals lived with normal light–dark cycles or in constant darkness (35). Feeding is therefore an ineffective zeitgeber for the SCN even in the absence of light, its primary zeitgeber. At the same time, feeding is a potent zeitgeber for the periphery regardless of opposing cues from the light-entrained SCN clock. Further studies confirmed that the food exerts a dominant zeitgeber effect on peripheral clock phase without affecting the SCN (36–40). Furthermore, phase advancing the light–dark cycle does not alter the phase of the liver clock if accompanied by a fixed restricted feeding schedule, and restricted feeding entrains the liver even in SCN-lesioned mice (37).

Nevertheless, the peripheral clock phase is still indirectly controlled by the light-entrained SCN. This is demonstrated by the fact that when feeding rhythms and the light phase are inverted, peripheral clock resetting is slowed by opposing signaling from the SCN. In nocturnal rodents who normally consume most food during the dark phase, restricting the feeding schedule to the day produces slow phase resetting of the liver and kidney to match feeding rhythms, with comparatively rapid resetting when returning from day to nighttime eating; this effect of slowed switching in the ‘wrong direction’ does not occur in adrenalectomized or glucocorticoid insensitive mice (41). That the master clock acts to counteract discordant entrainment of peripheral clocks by light phase feeding was confirmed by *in vivo* recording of *Bmal1*-driven luciferase activity in the hepatocytes of SCN-lesioned mice. After only 6 days of daytime restricted feeding, the hepatocytes in the SCN-lesioned mice had undergone a steady and complete phase shift to match the food intake rhythm, in contrast to the sham-operated mice where the hepatocyte clock had only partially phase-shifted this short time (42).

The differential responsiveness of tissues to a particular zeitgeber makes evolutionary sense because the component of an endogenous clock presented to natural selection is its phase relationship with respect to the environmental events it anticipates. As tissues become specialized in their function, the relevance of environmental cues as zeitgebers would be aligned accordingly. For example, in a metabolically active organ such as the liver, regularly timed food intake is an important event with which metabolic processes are synchronized. Among the peripheral organs studied in the literature, the liver clock phase shifts the most rapidly in response to restricted feeding (35), corresponding to its primary metabolic function. It is interesting that while SCN is not sensitive to time-restricted feeding, it does respond to long-term fasting and caloric restriction. The SCN phase shifts in response to caloric restriction (43) and synchronizes to a single hypocaloric meal given at the same time each day in constant darkness (44). Whereas, acute differences in food intake timing are not relevant zeitgebers to the SCN, perceived starvation certainly is.

## Circadian Disruption and Metabolic Dysfunction

Under normal physiological conditions, the light-driven SCN rhythm and feeding-driven peripheral rhythms are aligned. However, light generally has only indirect effects on peripheral clocks through signals from the SCN, and feeding behavior resets the phase of peripheral clocks without shifting the SCN clock phase, which produces the possibility of misalignment between the central and peripheral rhythms. The sustained discordance of feeding time with the activity phase governed by the central pacemaker was shown to result in peripheral uncoupling in rodents (35). As we will see, the modern food environment and socially enforced behavioral rhythms produce similar effects in humans today with profound metabolic consequences.

### Mouse Models

The causal relationship between the disfunction of the clock and metabolic disease is clearly demonstrated in the phenotypes of circadian mutant mice. It was first observed that deletion of *Bmal1* led to severe glucose dysregulation (45), and perturbation of metabolic homeostasis was subsequently found in mice mutated in other clock components. There is significant phenotypic variation depending on the clock component mutated, but what all have in common discernable metabolic dysregulation (46). It is likely that the clock function, rather than any other function of these particular genes, is at the root of the metabolic dysregulation because a similar result is observed repeatedly in the ablation of various clock components (18). The converse case of circadian disruption in models of metabolic disease is also observed: the loss of feeding rhythms precedes the development of obesity in leptin deficient *ob/ob* mice (47, 48) and in diet-induced obesity models (49, 50). Moreover, the effects of genetically “breaking” the clock by knocking out a key component in mice are in many cases similar to the effects of chronic stress on the circadian system through behavioral misalignment in humans.

### Humans

#### Shift Work

There is now a substantial body of evidence for the role of disrupted circadian rhythms in metabolic disease pathogenesis. Epidemiological studies on long-term shift workers were the first to demonstrate the connection between circadian misalignment (51) and metabolic disease (52–54). Mechanistic support in animal models of shift work was reviewed by Opperhuizen et al. (55). In a classic demonstration of the effects of misalignment in humans, a 10-day laboratory protocol forced desynchrony with a 28-h day, resulting in widespread alterations in the rhythms of plasma leptin, glucose, insulin, and cortisol levels (56). In 3 out of 8 subjects, just 10 days of misalignment induced a prediabetic state.

Indeed, acute circadian disruption in a laboratory setting reliably alters glucose metabolism and induces a diabetogenic state in humans [reviewed in (57, 58)]. In a randomized trial simulating night shift work, 3 days of a phase inversion (i.e., awake and eating during the inactive phase) showed significant impairment in insulin sensitivity and greater postprandial glucose rise in response to the same meal (59), and after 6 days of phase inversion, plasma proteomics revealed alterations in the rhythms of many proteins known to regulate glucose homeostasis, along with much higher postprandial glucose and insulin (60). The plasma glucagon rhythm was inverted to match feeding schedule, but importantly was also elevated under nighttime food intake, which itself is a risk factor for diabetes (61). Elevated glucagon is potentially explained by the stimulatory action of melatonin (which peaks at night) on pancreatic alpha cells (62). Finally, observational evidence supports that chronic circadian disruption impairs glucose homeostasis and increases disease risk, with shift work increasing risk of developing Type 2 diabetes (63, 64).

The composition of the diet and energy intake during night shifts is unlikely to be the major contributor to metabolic disruption. A recent survey of eating patterns in night shift workers showed that, except for slightly higher sugar and lower saturated fat consumption during the night shift, there was no difference in caloric intake or adherence to dietary guidelines compared with day shifts or days off (65). This minor shift toward carbohydrate intake compared with fat intake during night shifts is consistent with data showing that improperly timed feeding in mice resulted in inefficient energy utilization and greater reliance on carbohydrate oxidation (66), an unfortunate combination with the impaired glucose handling induced by circadian misalignment.

The greatest change in nutrient input during nightshifts though is in its timing, and this can in itself contribute to metabolic disruption. During nightshifts meals are spread out over the 24 h period, as compared to dayshift or off days where the fasting window generally approximates sleep time (65, 67). Therefore, like in laboratory simulations of shift work with controlled isocaloric meals, the main variable with respect to food intake is its timing with respect to the endogenous clock. Notably, the eating pattern of shift workers on their days off, where fasting duration approximates sleep duration, has also been observed in the general population. Data collected from a smartphone application showed that the majority of healthy adult participants ate at random intervals in a window of 14–15 h (68). The tendency in modern humans is to shorten the overnight fast by continuing consumption into the inactive phase of the circadian period; when food is constantly available, the only constraint on eating is to be awake. Impressively, even modestly condensing the feeding window to 10–11 h led to weight loss and lower plasma insulin levels, as self-reported through the application (68). The effects of meal timing and time-restricted feeding (TRF) are explored in greater detail in section Effects of Meal Timing.

#### Social Jetlag

Shift workers perhaps represent the extreme end of circadian misalignment, but they are not alone in their vulnerability to its deleterious effects. Constant food availability, reduced overall sleep, and longer active hours are common to the modern lifestyle [reviewed in (69)]. Many people experience a chronic mismatch between their endogenous circadian rhythms and socially dictated rhythms of behavior, such as those enforced by work or school start times. The discrepancy between internal and imposed rhythms can be quantified by the difference between the sleep midpoint on work and work-free days. This phenomenon, termed *social jetlag*, is experienced by as much as 87% of the day-working population (4). Distinct from shift work, social jetlag has also been independently associated with obesity (70), the risk of developing T2D (71), abdominal adiposity, and metabolic syndrome (72).

#### Common Disruptive Factors

At least three factors are common to the circadian disruption seen with shift work and social jetlag; (1) light exposure during the dark phase; (2) food intake during the inactive phase; (3) sleep disruption. Indeed, sleep duration alone is strongly associated with the development of metabolic syndrome and accounts for a large proportion of the cardiometabolic risk in shift workers (73). Sleep deprivation has also been shown experimentally to reduce insulin sensitivity (60, 74) and has been strongly linked to development of obesity (75, 76) and diabetes (77). Although the harms of sleep deprivation are manifold, these will not be discussed in detail in the present review. Sleep is not itself a zeitgeber, but rather an output of the SCN clock (78) as well as a modulator of responsiveness to zeitgebers such as light (79). Importantly, while sleep deprivation alone induces insulin resistance in controlled laboratory experiments, circadian misalignment causes additive impairment of insulin sensitivity even with sleep time kept constant at a meager 5 h (80). When considering circadian misalignment, it is what we do when awake at the wrong time, i.e., eating and light exposure, that matters for clock entrainment.

##### Light at night

As previously described, light is the most powerful zeitgeber of the master clock in the SCN. In a population of healthcare workers, the phase shift of the master clock after 3–4 consecutive nightshifts could be 71% accounted for by intensity of light exposure according to the individual's baseline phase response curve (81). The SCN clock in turn regulates system wide energy metabolism as well as activity and food seeking behavior in alignment with these light-entrained rhythms (82). This finding helps to explain why, independent of other lifestyle factors (e.g., sleep duration, physical activity, and smoking), light at night (LAN) has been correlated with the increased risk of developing obesity in humans (83) and has been shown to acutely induce glucose intolerance in rats (84).

##### Mistimed food intake

Mechanistic studies in animals point to shifts in food consumption as a potential underlying cause of this correlation between LAN and obesity. Mice exposed to LAN gained weight and had impaired glucose tolerance, despite equivalent calorie intake and daily activity, because their food consumption was redistributed into the rest phase (85). This mistimed food intake results in peripheral misalignment. After 8 weeks of a rotating light schedule consisting of 3 days of normal light/dark (L:D 12:12) followed by 4 days of reversal (D:L 12:12), mice lost daily rhythms in fuel utilization and energy consumption, leading to greater weight gain, elevated blood glucose and lipids, and hepatic steatosis (86). In sum, mistimed food intake can account for a large proportion of the deleterious metabolic effects of light-induced circadian misalignment.

Metabolomics studies support the strong effects of food intake on rhythmic metabolism in humans. In constant conditions of enforced posture, dim light, sleep deprivation, and hourly isocaloric meals, ~15% of the metabolites in saliva and plasma were found to have a circadian rhythm, with most in saliva being amino acids, and most in plasma being lipid metabolites (87). Among these were prominent rhythms in free fatty acids and triglycerides, which peaked during the light phase; since they were independent of feeding or rest-activity cycles, this suggested endogenous circadian oscillators control lipid metabolism. In contrast to the approximately 15% metabolites that were rhythmic independent of feeding rhythms, when participants were fed normal meals (i.e., three daily meals plus a snack), 60–70% of metabolites became rhythmic, and most of these retained rhythmicity during constant wakefulness (88). Thus, rhythmicity is gained in as much as half the human metabolome through rhythmic feeding, while sleep/wake rhythms have comparatively little effect.

It is worthwhile to note that it is possible some metabolite rhythms newly observed with meals are properly thought of as being disinhibited by the introduction of fasting periods in this schedule compared to the hourly meals in constant conditions. Nutrient-sensing pathways active during periods of fasting, such as AMPK and the Nampt/NAD+/Sirt1 feedback loop (89), are intertwined with the core clock feedback loop and thereby affect widespread outputs of circadian metabolic regulatory mechanisms (15). Constant conditions were used to observe free-running endogenous rhythms in the absence of zeitgeber cues, but both food intake and fasting can serve as cues to the clock. The light-entrained SCN clock is well-understood to have a different free-running period under constant dark (DD) or constant light (LL) conditions (90). Similarly, it would be worthwhile to investigate the rhythms in metabolites thought to be outputs of food-entrained peripheral clocks under constant fasting as well as constant feeding conditions.

Behavioral rhythms are the predominant drivers of metabolite rhythms, which is reflected by phase shifts in metabolite rhythms during shift work. Targeted plasma metabolomics showed phase shifts in 95% of rhythmic metabolites when measured under constant routine following 3 days simulated shiftwork, with many metabolites following the 12 h phase inversion of behavior (activity and feeding) from the preceding 3 days (91). In another metabolomics investigation of simulated shift work with a 10-h delay of behavior, 75% of oscillating plasma metabolites were phase-shifted with an average phase delay of 8.8 h, although there was high interindividual variability (92). Urine metabolite rhythms were also altered in night shifts, particularly in individuals with an early chronotype [defined by the midpoint of sleep; (93)], as was the plasma proteome (60). Importantly, SCN clock outputs (i.e., melatonin and cortisol) were consistently found *not* to be phase-shifted in laboratory-simulated shift work (91, 92, 94) or in most permanent shift workers (95), thereby producing the anticipated misalignment with the periphery. The timing of food intake is unquestionably a powerful stimulus for peripheral circadian entrainment, having the potential to cause misalignment of metabolic processes. Of the deleterious modern lifestyle factors, it is also the most amenable to intervention.

## Effects of Meal Timing

### Potential Benefits of Early Meals

Due to the differing responsiveness of tissue clocks to zeitgebers, it is possible for phase misalignment to occur when these cues are not presented together at their usual times. This is one way by which food intake during the dark phase can result in systemic metabolic dysregulation. An additional mechanism by which food intake timing can alter metabolic function comes from the circadian regulation of homeostatic responses to nutritional challenge. The same meal consumed at different times of day can produce distinct responses because of circadian variations in energy uptake and utilization (58, 96), and the master clock of the SCN controls the endocrine response to nutrient intake. For instance, the same meal eaten at 8 a.m. instead of 8 p.m. produced greater postprandial epinephrine and norepinephrine and less acylated ghrelin (97). These observations indicate that because of the circadian regulation of food intake response, the timing of the feeding period is a decisive factor in nutrient handling.

In this respect, recent evidence has reinforced the advice of Maimonides, the Jewish philosopher and doctor (also called Rambam, ca.1135): “Eat like a king in the morning, a prince at noon, and a peasant at dinner.” Indeed, those who consume most of their daily calories at dinner are at the increased risk of developing obesity and metabolic syndrome (98, 99). In a 20-week weight loss trial, subjects with an overall food intake later in the day lost less weight and had slower weight loss despite similar caloric intake, energy expenditure, and sleep duration (100). Similarly, a randomized control trial of calorie timing during weight loss found the “big breakfast group” (200/500/700 kcal rather than 700/500/200 kcal in three daily meals) lost more weight and inches around their waist. The big breakfast group also had a greater decrease in fasting glucose, insulin, and insulin resistance, while reporting greater satiety, lower hunger, and having concordantly lower ghrelin levels (101). Moreover, skewing energy intake toward breakfast rather than dinner in patients with T2DM reduced postprandial hyperglycemia and average glucose while increasing insulin and incretin levels (102). Taken together, caloric intake weighted toward the beginning of the active phase (i.e., a big breakfast and small dinner) may confer a metabolic advantage, especially in the context of weight loss, by coordinating food intake to coincide with the circadian timing of nutrient handling.

### Entrainment of Peripheral Clocks to Meal Timing

Similar benefits of early meal timing have also been observed in healthy subjects. In a highly controlled in-lab crossover experiment, healthy young men ate identical meals at 5-h intervals beginning either 0.5 h (early schedule) or 5.5 h (late schedule) after waking. The participants were acclimated to early meals for 3 days and then switched to late meals for 6 days. The circadian rhythms of the participants were measured after each meal schedule in a 37-h constant routine (i.e., no sleep, dim light, and hourly isocaloric meals) to observe the effects of the eating schedule without acute effects of consumption. Previous exposure to the late meals decreased average glucose without altering insulin rhythms, corroborating the benefits of earlier meals observed in the context of weight loss (101). Glucose rhythms were also delayed (6 h) in accordance with meal timing (delayed 5 h) in the prior days without changes in the levels of central clock markers, indicating the entrainment of peripheral clocks but not the SCN (103). Indeed, a smaller delay (~1 h) was observed in adipose tissue PER2, serving as proof of principle that meal schedule can entrain the peripheral clock phase in humans. A recent study in mice suggests that postprandial rises in insulin and IGF-1 may directly induce PER protein translation in peripheral tissues (40).

Studies in rodents suggest that food entrainment of the adipose clock, as compared to the more rapidly adapting liver clock, may require longer-term exposure to the new meal schedule (104). The rapid entrainment of the liver clock could explain the disproportionate effect of meal timing on glucose compared to lipid levels, the latter having been unaffected by 6 days of entrainment to earlier meal timing (103). Lipid levels are known to be under circadian regulation in humans, and a large proportion of the circadian metabolome under constant conditions is comprised of lipid species (87). However, their regulation involves multiple peripheral tissues, such as adipose, whose clock may be entrained gradually. On the other hand, glucose homeostasis has been demonstrated in mice to be the direct output of the liver-independent circadian clock. Koronowski et al. (10) examined the circadian metabolome and transcriptome of the liver in the absence of all other tissue clocks using a hepatocyte-specific reconstitution of Bmal1 in whole-body Bmal1 knockout mice (Liver-RE). The reconstituted livers showed the restoration of 10% of transcript and 20% of metabolite oscillations, and glucose metabolism pathways were among the most represented in these independent hepatocyte clock outputs. The rhythms in hepatic glycogen synthesis and turnover were almost completely restored as well as the rhythmic recruitment of Bmal1 to the rate-limiting enzyme *Gys2* (glycogen synthase 2). Moreover, regulation of hepatic *Glut2* by BMAL1 drove active phase peaks in glucose levels that were otherwise absent in the full knockout. Previous studies on mice showed that the liver was the most rapidly entrained by feeding time (35) and that its clock autonomously drove rhythmic plasma glucose levels (10). Together, studies in mice have shown that the liver clock is most sensitively entrained by feeding time (35, 104) and autonomously drives rhythmic plasma glucose levels (10), which could explain the relative sensitivity of human plasma glucose but not lipid rhythms to the acute changes in feeding schedule observed by Wehrens et al. (103).

### Components of Meal Timing

**Figure 3:**
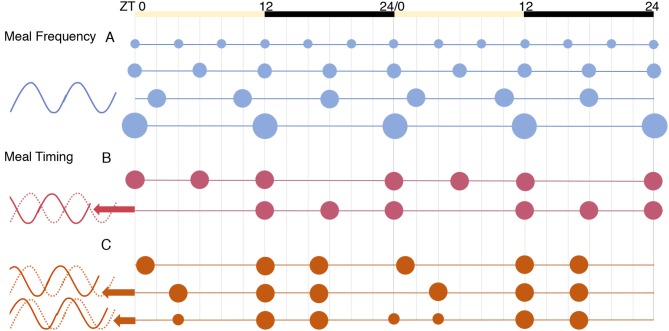
The effects of meal frequency vs. timing on peripheral tissue clocks [from results of (105)]. The phase of Per2 expression was similar in all conditions for the tissues tested: liver, kidney, and submandibular gland. **(A)** Meal frequency, spreading calories equally into 2, 3, 4, or 6 meals per day, did not alter clock phase. **(B)** Daytime (inactive phase) feeding significantly advanced phase. **(C)** Using a three-meal schedule designed to mimic human eating habits, a “late dinner” meal (in the early inactive phase) caused a dramatic phase advance, which was partially reversed by splitting the dinner meal to include a “pre-dinner snack”.

The timing of meals as a potential zeitgeber is comprised of multiple distinguishable variables: meal frequency, the length of eating or fasting window, and its timing with respect to the day/night cycle. The effect of meal frequency vs. timing on the core clock was explored by Koruda et al. (105) using *in vivo* recording of Per2 activity in the kidney, liver, and the submandibular gland of circadian reporter (Per2:Luciferase) mice. Meals of varying frequencies (2, 3, 4, or 6 meals) given at equal intervals throughout the 24 h period did not affect the phase of clock genes in these peripheral tissues [Figure 3A and (105)]. In contrast, a constant frequency of three meals per day given in different temporal patterns resulted in significant changes in the clock phase. A “late dinner” (ZT4) caused a dramatic phase advance in peripheral clocks, which was partially reversed when the dinner was split into two smaller meals to include a “pre-dinner snack” (ZT0) in the period before the late dinner (Figure 3C). Viewed from another perspective, the late dinner created a shorter fast before breakfast and a longer fast after lunch, the latter being filled when a pre-dinner snack was added. This illustrates the importance of feeding intervals or periods of fasting in peripheral clock entrainment, which is a plausible mechanism given the interaction between the core clock and nutrient-sensing pathways (e.g., AMPK) that are activated during fasting (9).

The importance of the fasting period is relevant to the interpretation of research in chrono-nutrition. For instance, a recent observational study found no association between BMI and the timing of meals in the self-reported data of 125 adults collected from a smartphone app (106), a result that at first glance is contradictory. However, the food timing variables analyzed were the “time of first eating episode” and “time of last eating episode,” neither of which by itself is indicative of fasting period. As a positive example of the relevance of fasting period, a randomized controlled trial in patients with T2DM found advantages of two meals/day compared with an isocaloric regimen of six meals/day. Eating only breakfast and lunch resulted in reduced body weight, fasting glucose, glucagon, and increased insulin sensitivity (107). From the above study in rodents (Figure 3), these results were likely due to the extended fasting window rather than reduced meal frequency. Observational work in humans is also in agreement with this. Individuals who ate one or two meals per day showed a BMI reduction over a one-year period, while increased BMI was observed in those who ate more than three meals a day; a greater reduction in BMI was observed in individuals who fasted at least 18 hours overnight compared to a medium overnight fast of 12–17 hours, as well as in those who consumed more calories early in the day (108). The authors concluded that a practical strategy for BMI reduction would be to consume the majority of calories early in the day and extend the overnight fast to 18 hours. Thus, both the length of the fasting period and the timing of meals with respect to the circadian day appear to be decisive in the maintenance of a healthy weight.

### Time-Restricted Feeding

The practice of limiting daily food intake to a finite time window, thereby extending the overnight fast, is referred to as time-restricted feeding (TRF). This mode of eating has received considerable attention in recent years as a strategy for aiding weight loss and improving metabolic health (109) and as an alternative to caloric restriction to promote longevity (110). Despite isocaloric intake, numerous benefits are observed simply by restricting the intake window. However, important questions remain regarding (1) the length of the feeding window permitted required to observe these benefits, and (2) the timing of the feeding window with respect to the 24-h day. Although controlled comparisons of early and late TRF in humans have not yet been conducted, the inference drawn from rodent models is clear: it is imperative that restricted feeding windows occur during the active phase. Mice in inactive phase TRF were hyperphagic and gained more weight than the active-phase restricted feeders within only 9 days; they showed reduced metabolic flexibility with greater reliance on carbohydrate, and as previously outlined, peripheral clock gene expression in metabolic organs was perturbed with near complete phase inversion in the liver and strongly dampened rhythms in epididymal adipose, skeletal muscle, and the heart (39). In rats, active phase TRF improved whereas inactive phase TRF worsened glucose tolerance (111), and inactive phase TRF caused desynchrony of muscle and liver clocks (112).

The shifts in peripheral clocks were both consequences and causes of metabolic dysregulation under rest-phase feeding (113), producing a vicious circle of misaligned processes. By contrast, active phase TRF does not appear to alter the peripheral circadian phase compared with *ad libitum* (AL) feeding (114), somewhat expected given that AL fed mice consumed ~80% of their food during the active phase when fed normal chow (35). The lack of phase shift during active phase TRF could also reflect the nature of the clock's response to zeitgebers: the same input produces phase changes that vary in magnitude and direction depending on time of day when exposure to the zeitgeber occurs (115). The master SCN clock is well-understood to be responsive to light entrainment during the dark phase, whereas the response to non-photic cues, such as arousal, is greater during the inactive phase (116). The presence of a zeitgeber outside its usual time induces phase shifts; peripheral clocks are therefore likely to have a characteristic phase response curve for food entrainment with greatest response coinciding with the inactive phase.

#### Early Time-Restricted Feeding (eTRF)

In humans, restricting food intake to early in the day is associated with reductions in bodyweight, but the mechanism of this association was not agreed upon until recently. Some researchers have speculated from work in rodents that it was the result of greater energy expenditure early in the day (117). Others reported reduced caloric intake due to decreased appetite during early meals. From a clinical standpoint, natural appetite reduction is, of course, a viable method of weight loss. The findings in a recent series of controlled feeding studies in humans support that a natural reduction in food intake is the primary mechanism behind weight loss during early time-restricted feeding (eTRF). In three trials, the participants in the eTRF groups ate during a 6-h window beginning at 8:00 or 9:00 a.m., and the controls ate an equal number of calories during a 12-h window beginning at the same time. After 5 weeks on isocaloric eTRF, men with prediabetes had improved insulin sensitivity accompanied by increased pancreatic ß-cell responsiveness and lower fasting glucose, blood pressure, and oxidative stress, as well as reduced subjective hunger (118). However, there were no differences in body weight compared to the controls; weight loss did not occur in early restricted feeding alone when caloric intake was matched, although metabolic improvements were observed.

This conclusion was supported by the results of two shorter term trials of eTRF. Overweight adults were randomized to 4 days of eTRF and studied using whole room indirect calorimetry on the final day. The results confirmed that energy expenditure was not significantly affected by the feeding time (119). The thermic effect of food was greater in the morning eaters, which was consistent with the results of previous work (59, 120), however, this effect was predicted to increase energy expenditure by only 20–40 kcal/day. This daily change is small, but over time it could be clinically significant. Importantly, other beneficial effects that produce weight loss in practice were observed in the early TRF group: levels of the satiety hormone peptide YY (PYY) and subjective “stomach fullness” were higher, while ghrelin, perceived “hunger” and the “desire to eat” were all significantly reduced (119). Metabolic flexibility, that is the ability to switch between carbohydrate and fat oxidation, was also increased in the TRF group, who more effectively burned fat during their fasting period. In a final trial using the same eTRF schedule, this eating pattern was shown to decrease mean 24-h glucose and glycemic excursions (121). Intriguingly, many of the clock genes appeared to be upregulated in the eTRF group, although the interpretation of rhythm was not possible because only two timepoints were sampled. Taken together, the results of these trials supported that eTRF can cause weight loss primarily through reducing hunger and partly through minor increases in the thermic effect of food, while also independently improving metabolic parameters. Human clinical trials aimed at better defining the contribution of endogenous circadian physiology to these effects are currently underway as part of the Big Breakfast Study (122).

#### Time-Restricted Feeding in Shift Workers

In addition to weight loss, a highly promising application of TRF is to maintain the cardiometabolic health of shift workers. The predominant effect of properly timed feeding in the presence of the factors of light exposure, sleep disturbance, and activity during the rest phase was clearly demonstrated by Salgado-Delgado et al. (123). Shift work was simulated by keeping rats on slow rotating wheels that forced wakefulness and a low level of activity from 9:00 to 17:00 (ZT2-ZT10), which is the inactive phase in nocturnal rodents, on weekdays for 5 weeks. On weekends, all rats were left undisturbed in their home cages with AL food access. During the work week, shift workers and controls were assigned to AL feeding, active phase TRF, or inactive phase TRF. Unsurprisingly, the AL-fed shift workers ate most of their food during the inactive phase when they were kept awake. They and the inactive TRF groups gained more weight and had greater visceral adiposity compared with the AL controls, active phase TRF controls, or active phase TRF shift workers, despite similar food intake across all groups. Even though the work schedule disrupted sleep and activity patterns, only *mistimed feeding* explained the differences in weight and fat gain. The corticosterone rhythms remained unchanged, corroborating that SCN clock outputs are resilient to behavioral feedback, which contributes to internal desynchrony during shiftwork. However, disturbances in glucose and TAG rhythms and the accumulation of abdominal fat were all prevented when shift work was combined with active phase TRF. Similar protection by active phase TRF is also observed in simulated jetlag. Mice exposed to a 6-h phase advance in the light/dark cycle twice weekly with AL food access became overweight despite isocaloric intake, but this was prevented by active phase TRF (124).

## Diet Composition

In addition to the timing of food intake, the composition of the diet alters peripheral circadian alignment and acutely induces phase shifts. An important mechanism by which this occurs is through feedback from altered behavioral rhythms in feeding and activity. Other mechanisms include impaired or enhanced inter-tissue communication as well as interactions of the core clock with particular nutrient-sensing pathways. The following section focuses on the effects of the well-studied high-fat diet and the increasingly studied ketogenic diet on circadian metabolism.

### High-Fat Diet

The modern obesogenic food environment is most commonly modeled in animals with the high-fat diet (HFD). Mice given AL access to the HFD reliably develop obesity and a phenotype similar to the metabolic syndrome in humans, characterized by insulin resistance, hepatic steatosis, hypercholesterolemia, and dyslipidemia (125). As also occurs in humans, this metabolic disruption is preceded by circadian disarrangement.

#### Altered Rhythms in Feeding Behavior

In animals, an immediate alteration in rhythmic behavior is observed upon the start of the HFD. Whereas, normal chow fed animals are active and consume ~80% of their food during the dark period, HFD fed mice become hyperphagic and eat continuously through the inactive phase, such that total food intake becomes indistinguishable between day and night (49, 104). The rapid onset of behavioral arrhythmicity indicates that it is likely to occur by a mechanism independent of clock gene regulation, which would take longer to adapt. Highly palatable foods or particular nutrient compositions can directly and rapidly signal to orexigenic centers (126). The HFD in particular directly affects appetite regulating centers in the arcuate nucleus as well as the regions associated with hedonic/reward stimulation that influence food-seeking behavior (127–129). It appears that the homeostatic feeding circuits acutely responsive to the HFD are independent of clock regulation. One week of HFD did not alter clock gene expression in the SCN, arcuate nucleus, or pituitary (104). This is despite reciprocal connections between the arcuate and SCN (130). Moreover, 6 weeks of HFD did not alter clock gene expression in the mediobasal hypothalamus (49), a region containing AgRP neurons known to possess an autonomous clock controlling the rhythms of hunger and satiety (131). The HFD therefore appears to acutely alter feeding behavior by clock-independent central mechanisms.

Although the rapid shift in feeding behavior upon beginning high-fat feeding is likely to be clock-independent, the HFD does also interfere with the master clock in the SCN. As compared to normal chow (NC)-fed animals, in those fed the HFD the induction photic entrainment in the SCN (marked by *c-fos*) is impaired, as is locomotor activity phase resetting, and re-entrainment following “jetlag” (a 6 h advanced) (50). Interestingly, hypocaloric feeding also alters photic entrainment (132, 133), but in contrast to the HFD, enables a more rapid phase shift response to light (134). In summary, diet composition can alter photic entrainment of the SCN, but the most important effects of the HFD on the circadian system are likely to occur indirectly through phase shifts in peripheral clocks in response to altered feeding behavior.

#### Altered Metabolic Rhythms in Liver

Shortly following the altered feeding rhythm, the clock genes of peripheral tissues are altered in amplitude and phase. The liver clock is the most widely studied and, as previously mentioned, perhaps the most sensitive to feeding rhythms. Long-term HFD feeding was shown to dampen hepatic clock gene expression (135, 136). After just 1 week on HFD, the hepatic clock was found to phase advance by 5 h (104). A similarly sized phase advance of 4 h was confirmed by Branecky et al. (137). Interestingly, they found that similar to the liver's rapid response at the beginning of HFD, it rapidly reverted to its normal phase within 7 days of the return to normal chow. This response lagged behind the feeding rhythms, which were restored within 2 days. As the changes in amplitude and phase are consistently found to occur after the changes in feeding rhythm, they are likely to be the result behavioral feedback. Diet composition may act as a zeitgeber in the liver by altering food intake timing through initially clock-independent mechanisms.

The phase advance in the liver clock is comparatively mild compared with the large-scale changes to downstream clock-controlled genes (CCG) observed with the HFD. In a comprehensive study of the effects of HFD compared with normal chow (NC) on the circadian transcriptome and metabolome in mouse liver, Eckel-Mahan et al. (136) found that large proportions of all oscillatory metabolites and transcripts either gained or lost rhythms under the HFD. Moreover, most of those that were oscillatory under both diet conditions exhibited phase shifts and overall advance under high-fat feeding. Another common effect of the HFD was to reduce the amplitude of oscillating metabolites and transcripts, and the transcripts that lost oscillations on the HFD had a robust peak at ZT8, coinciding with the greatest activity of CLOCK:BMAL1 at target genes. For instance, during NC feeding, the levels of NAD+ oscillated because of the CLOCK:BMAL1 regulation of *Nampt* which encodes the rate-limiting enzyme in the NAD+ salvage pathway. The HFD impaired CLOCK:BMAL1 chromatin binding to this and other metabolic CCGs, leading to the loss of oscillation in this key enzyme and the flattening of hepatic NAD+ levels (136). In other cases, the HFD produced *de novo* oscillation of the metabolites and transcripts encoding their regulatory enzymes, such as in the methionine cycle. These *de novo* oscillations appeared to be downstream of the oscillations in the nuclear accumulation of transcription factors outside the core clock, especially PPARγ and SREBP-1. Because of the relation between the methionine cycle and epigenetic methylation reactions (138), its newly oscillatory status may be one mechanism in the large-scale reprogramming observed under an HFD. Importantly, many of these changes were observable after an acute HFD exposure of just 3 days and were reversed with 2 weeks on NC, confirming these effects were the result of the HFD rather than diet-induced obesity (136).

Corroborating and expanding on these results, the HFD was again recently shown to induce the rhythmic binding of non-core clock transcription factors to metabolic target genes in the liver, including SREBP-1. Guan et al. (139) found that rhythmic lipogenesis under SREBP-1 control produced endogenous ligands for PPARα, which then activated rhythmic fatty acid oxidation. As is usual under high-fat feeding, the mice developed insulin resistance, hyperlipidemia, and fatty liver. The latter condition is targeted in humans by PPAR agonist drugs. Impressively, treatment with the PPARα agonist in the HFD fed mice at the peak of its activity (ZT8) resulted in a greater reduction of hepatic lipid accumulation and serum triglyceride levels than when it was given at the nadir of PPARα activity (ZT20) (139). This strategic administration of drugs in coordination with circadian rhythms is an example chronotherapy, which is a promising direction for translational work. It is known that lipid profiles in humans have a robust circadian rhythm (88). There is also some evidence that levels of clock genes in circulating monocytes, particularly the PERs, are altered in switching from a high-carb/low-fat to a high-fat/low-carb diet (140). In this study, however, the samples were taken only at three timepoints within an 8-h period, which was insufficient to extrapolate 24-h oscillations. The effects of dietary fat on clock gene expression and circadian regulation in humans will require further investigation.

#### Impaired Inter-Tissue Communication and Misalignment

In addition to altering the liver clock and causing widespread alterations in rhythmic metabolism, the HFD impairs inter-tissue communication, thereby further exacerbating the misalignment of circadian rhythms between key metabolic tissues. For instance, adiponectin from mature adipocytes regulates hepatic lipid metabolism by activating AMPK, leading to the phosphorylation and inactivation of the fatty acid synthesis enzyme ACC, the activation of PPARα to increase FA oxidation, and the potentiation of insulin signaling to inhibit gluconeogenesis (141). In the livers of mice fed the HFD, rhythms in AMPK and ACC transcripts were abolished, and a 3-h phase delay and dramatic dampening were observed in key components of the adiponectin signaling pathway (AdipoR1, Pepck, and PPARα) as well as the core clock gene Per1 (141). A similar phenotype during HFD feeding was observed in skeletal muscle and adipose (142). In addition to adiponectin, normal daily rhythms in plasma insulin, ghrelin, and leptin have been shown to be disrupted in rats fed an HFD (143).

Along with endocrine system interference, the HFD also directly misaligns tissues by differentially regulating their clocks. The HFD causes rapid phase shifts in the liver within 1 week, whereas the clocks of the lung, spleen, aorta, gonadal white adipose were unchanged in phase (104). It is possible that these tissues rely less on food intake as a zeitgeber, or that they adapt more gradually to HFD exposure. Whether the liver responds more strongly or just more rapidly to the HFD, this discrepancy in responsiveness is important because the liver then quickly becomes misaligned with other tissue clocks.

The induction of peripheral misalignment by HFD was strikingly demonstrated in recent circadian metabolomics studies. Abbondante et al. (144) showed that a large proportion of serum metabolite rhythms are lost under the HFD and become uncoupled from liver rhythms. Dyar et al. (145) expanded this finding, performing metabolomics on serum, liver, white and brown adipose tissue, muscle, the medial prefrontal cortex (mPFC), SCN, and sperm, following either HFD or NC feeding. They found tissue-specific alterations in circadian metabolism that caused the large-scale disruption of the temporal cohesion between the tissues. Positive and negative correlations in time of tissue metabolites are indicative of shared metabolic networks and the temporal gating of incompatible metabolic processes, respectively. The major source of metabolite correlations on NC were through those circulating in serum. An astounding 98% of these metabolite correlations were lost in serum under the HFD, in addition to 74, 39, and 34% in brown adipose tissue (BAT), muscle, and liver, respectively (145). Lipids in particular were highly correlated in time under NC and they lost this inter-tissue alignment under the HFD.

Similar untargeted metabolomics were undertaken in human blood samples and muscle biopsies from overweight/obese men after 5 days of a high-fat or high-carbohydrate diet [65/20/15% vs. 15/20/65% calories from carbohydrate/protein/fat, respectively; (20)]. Unfortunately, however, samples were taken at 7:30 a.m. in the fasted state and at 7:30 p.m. after dinner, making it impossible to separate the effect of time of day from acute effects of the evening meal; indeed, among the metabolites heightened in the evening (after dinner) on the HFD were fatty acids and ketone bodies, while xenobiotics that are known food additives were higher in the evening on both diets. The effect of diet composition on the human circadian metabolome requires further investigation.

### The Ketogenic Diet

The term HFD has been used to refer to rodent chow of varying compositions, but a typical diet used to model human obesity is by calorie 45% fat, 15–20% protein, and 35–40% carbohydrate (146). Hence, the term HFD is somewhat of a misnomer that perhaps more accurately designates a high-fat, moderate-carbohydrate diet. This is to be distinguished from the even higher fat and very low carbohydrate ketogenic diet (KD). Ketogenic rodent chow is nearly devoid of carbohydrates; it is comprised of more than 90% fat and the remainder is largely protein (147). In humans, a KD typically restricts daily carbohydrates to under 50 g per day (148). Whereas, the HFD is used experimentally to induce obesity and metabolic disease, the KD is under investigation because of its therapeutic potential to ameliorate these states (149). Whereas, the HFD induces hyperphagia, appetite inhibition is thought to be an important part of KD efficacy (148). Similarly, the interaction between the KD and the circadian clock is quite distinct from the HFD case.

The KD drives metabolism toward the pathways induced under fasting or caloric restriction without the need to restrict energy intake. Gluconeogenesis, fatty acid oxidation, and ketogenesis are upregulated, while glycolysis and *de novo* lipogenesis are inhibited (150). The circulating levels of ketone bodies in healthy humans have long been recognized to follow daily rhythms, with overall higher levels occur during carbohydrate restriction as in the KD or in fasting (151). The induction of ketogenic genes in the liver during fasting is controlled by the mTOR–PPARα axis (152), and the PPAR family is known to be circadian-regulated (17, 153). Moreover, the hepatic expression of the clock component Per2 was shown to be necessary in ketogenesis. Per2 is a direct regulator of *Cpt1a* expression, a rate-limiting enzyme that transfers long-chain fatty acids to the inner mitochondrial membrane for ß-oxidation, and is an indirect regulator of *Hmgcs2*, the rate-limiting enzyme for ketogenesis from the resulting acetyl-CoA (154). The KD may also increase transcriptional activation of CCG's by the CLOCK:BMAL1 complex (peaking at ZT8-12); the KD diet has been shown to upregulate the clock output gene *Dbp* in the liver, heart, kidney, and adipose tissue (155).

The circadian transcriptome was recently analyzed in the liver and intestine (ileal epithelia) of mice fed the KD or isocaloric quantities of NC, revealing large-scale alterations in oscillating transcripts (147). In contrast to the HFD, which mildly dampened clock gene expression (136), the amplitude of clock genes were either unaltered or slightly increased under the KD. The rhythm in the respiratory metabolism was abolished by KD as it was in the HFD (147). The rhythm in respiratory metabolism was abolished by KD as in the HFD. An arrhythmic respiratory exchange ratio (RER) is considered evidence of metabolic impairment (156), as the body is unable to efficiently switch from burning carbohydrate to burning fatty acids during the overnight fast. However, in the context of the KD, this flattened RER is an expected outcome given that fatty acids are the predominant fuel available. Indeed, the RER remained at ~0.7 on the KD, indicative of purely fat rather than carbohydrate oxidation (147).

**Figure 4:**
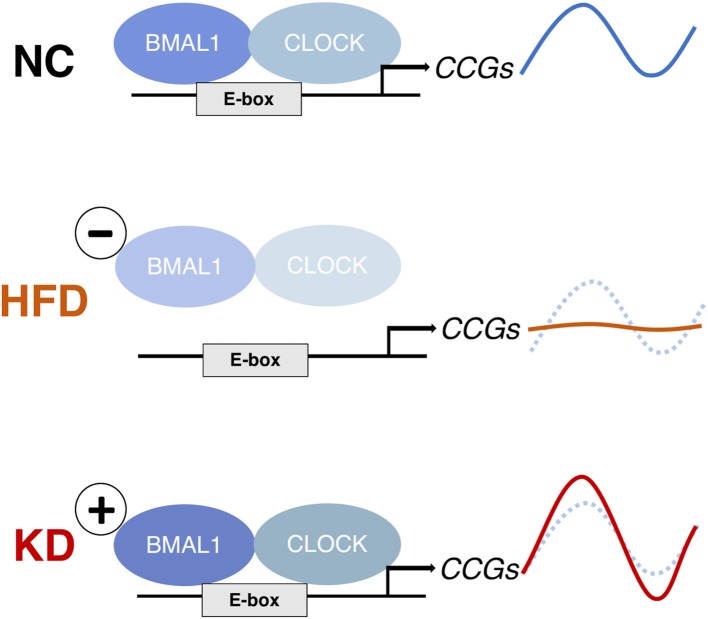
The effects of a high-fat diet (HFD) and ketogenic diet (KD) on clock-controlled gene (CCG) expression in mouse liver. The HFD abolishes rhythms in CCGs by reducing CLOCK:BMAL1 chromatin binding (136), whereas the KD increases rhythmic CLOCK:BMAL1 binding and thereby CCG expression amplitude (147). Both diets induce *de novo* rhythms in other metabolic genes because of the rhythmic nuclear accumulation of non-clock transcription regulators (PPARg and SREBP-1 in liver under HFD; PPARα in intestine and rhythmic histone deacetylation by serum BHB under KD).

Similar to the case of the HFD, the greatest alteration in the liver was not at the level of the clock genes themselves but in the chromatin recruitment of CLOCK:BMAL1 to clock-controlled genes (CCGs). However, the overall effects were opposite (Figure 4). In the HFD, the occupancy of CLOCK:BMAL1 at target gene promoters was attenuated, causing blunted oscillations of CCGs (136). In contrast, the KD increased the amplitude of clock target genes, including *Dbp* and *Nampt*, following greater Bmal1 recruitment to these sites at the critical timepoints. This KD-induced amplitude increase was not seen in *Clock-*mutant mice, confirming it is the output of the clock (147).

In the intestine of KD fed mice, the dietary intake of fatty acids induced rhythmic nuclear accumulation of PPARα and the expression of its target genes (147). Of all oscillating transcripts in the intestine, ~20% were expressed in phase with the nuclear accumulation of PPARα, including *Hmgcs2*. Corresponding to the HMGCS2 induction were oscillations of the ketone body ß-hydroxybutyrate (ßOHB) in the gut and serum. ßOHB has received recent attention as a signaling molecule with histone deacetylase activity (151), and it appears to also be a cofactor in a recently defined epigenetic marker, histone b-hydroxyl-butyrylation, which is associated with active gene expression (157). Therefore, transcriptional reprogramming under the KD may partly be through chromatin remodeling by ßOHB. Whether the overall effect of this large-scale remodeling is beneficial is not yet clear; however, a positive effect of intestinal ßOHB in maintaining the stemness and regenerative capability of intestinal stem cells was recently identified (158).

## Interaction Between Diet Composition and Timing

Experiments using circadian mutant mice fed the HFD illustrate the interaction between circadian clock function and the response to different diet compositions. The RAR-related orphan receptor alpha (RORα) is a nuclear receptor that functions as a ligand-dependent transcriptional factor in lipid metabolism and in circadian regulation (159). The natural loss of function occurs in *Ror*α mutants, which are called Staggerer mice (RORα^*sg*/*sg*^ mice). These mice exhibit a lean and dyslipidemic phenotype when fed NC. When fed the HFD, they are resistant to weight gain and hepatic steatosis, although they also develop particularly severe atherosclerosis (160). This finding attracted attention to RORα and other circadian nuclear receptors (Reverb's) as drug targets for metabolic disease (161). Recent work has suggested that RORα agonists may be effective in preventing hepatic steatosis (162), and liver-specific *Ror*α deletion correspondingly worsens hepatic steatosis (163). However, RORα appears not to alter the susceptibility to obesity and metabolic disease in mice fed a Western-style diet [40% fat, 40% carbohydrate; (164)]. Thus, the particular phenotype of circadian mutant mice is dependent on diet composition.

Further illustrating this interaction between the circadian clock and diet composition, some circadian mutants have strikingly different phenotypes on NC compared to the HFD. The phenotype of Per2–/– animals fed NC is of lower bodyweight and fat due to overaction of PPARγ target genes (*Ucp1, Cidea*) in adipose (165), but, when fed HFD the Per2 knockouts are more prone to gain fat than WT littermates. Similarly, Cry1–/– Cry2–/– double knockout mice have lower body weight and adiposity on NC because of increased energy expenditure, but when fed the HFD, they gain fat dramatically more quickly than WT mice despite being comparatively hypophagic, in part due to hyperinsulinemia-stimulated lipogenesis (166). It is interesting that in both cases, the mutant mice exhibited disrupted feeding rhythms even on NC, but a disrupted core clock and feeding rhythm were not sufficient to produce weight gain. Instead, they strongly predisposed the mice to diet-induced obesity.

### Preventing Diet-Induced Obesity Using Time-Restricted Feeding

Both eating at the wrong time and the HFD independently lead to internal misalignment between metabolic organs (84, 167). The HFD also acutely alters food intake timing, and this appears to be at the root of some of its adverse effects on circadian metabolism. The time-restricted feeding (TRF) of HFD within 8 h during the active phase, despite isocaloric intake compared with AL-fed controls, protected mice against diet-induced obesity (DIO) and related metabolic dysfunction (117). Similarly, mice on a 12-h active phase TRF were able to maintain normal metabolic parameters on both low- and high-fat diets (168). The combination of this obesogenic diet with active phase TRF also restored the rhythms in liver clock gene expression (169) and reduced plasma insulin, leptin, and proinflammatory cytokines, suggesting protection against systemic inflammation (170).

Active phase TRF acts as a protective buffer against an array of nutritional challenges. Although the HFD is the most common diet used to induce obesity in rodent studies, it is one among multiple experimental diets that model the energy-dense and processed foods eaten by modern humans and produce characteristic metabolic disease. For instance, a high-fructose diet causes insulin resistance, cardiac damage, and hepatic steatosis (171, 172). To address the efficacy of TRF to combat weight gain and disease on different obesogenic diets, one large mouse study examined three different experimental diets: a high-fructose diet (13% fat, 60% fructose); a high-fat and sucrose diet (32% fat, 25% sucrose); and the HFD (62% fat, 20% carbohydrates); a control chow (13% fat, 59% carb). They found that restricting food access to 8–9 h during the active phase effectively prevented or even reversed the already established obesity and metabolic dysfunction caused by any of these diets when fed AL, and this was accompanied by the restoration of temporal dynamics in several key metabolic pathways (173). Relevant to humans, this active phase TRF provided effective protection against diet-induced metabolic disease even when mice fed freely on weekends.

To get the protective benefits of TRF it is imperative that the feeding window occur during the active phase. In fact, restricting access to the HFD to the “wrong time” (rest phase) caused a 2.5-fold greater weight gain compared with active phase feeding (174). Timing the food intake with the active phase appears to confer a metabolic advantage because physiological systems are more prepared for the nutritional challenge. For example, in the response to a high-fat meal at the beginning of the active period fatty acid oxidation was shown to be increased, but this metabolic flexibility was lacking in the response to the same high-fat meal at the end of the active phase (168). Over time, improperly timed feeding can promote metabolic disease independently of the daily total number of fat-derived calories consumed (168). In addition, inactive phase TRF of either the HFD or NC for 6 days almost completely inverted the liver clock's phase (135), causing misalignment with other peripheral clocks that were not as responsive to this zeitgeber. The same peripheral misalignment was observed in mice on inactive phase TRF of a high-fat high-sucrose (HFHS) diet, along with hyperphagia due to central leptin resistance (175). Collectively, the inactive phase feeding of an array of diets (NC, HFD, or HFHS) impairs nutrient handling, induces peripheral clock misalignment, and disrupts signaling between metabolic tissues.

The HFD and other obesogenic diets interfere with normal feeding rhythms, but mice consuming isocaloric amounts of obesogenic diets stay slim and healthy as long as consumption is confined to the active phase. Mutations of clock genes also result in disrupted feeding and activity rhythms in gene-specific metabolic phenotypes. As the results of previous work have shown, the susceptibility of circadian mutants to obesity and various metabolic diseases depends on the composition of the diet they are fed in interaction with their particular genotype. Significantly, restoring feeding rhythms with time-restricted feeding in circadian mutant strains that are normally susceptible to HFD-induced obesity (a liver Bmal1 KO; liver Rev-Erb α/β DKO, and full-body Cry1/Cry2 DKO) was sufficient to prevent DIO and resolve some genotype-specific metabolic abnormalities (176). This result suggests that the metabolic disruption in circadian mutant animals and animals fed the HFD secondary to their altered feeding rhythms, and that imposing an active/inactive phase rhythm of feeding/fasting is sufficient to prevent the metabolic disease phenotypes of these circadian mutants. Such an interpretation is further supported by a recent study in mice with SCN-targeted ablation of Bmal1. In constant darkness, these mice develop arrhythmic activity, feeding, and peripheral clock gene expression (in the liver, pancreas, and adipose). With this arrhythmicity comes increased adiposity and impaired glucose tolerance. However, TRF was sufficient to restore peripheral tissue rhythms, body weight, and glucose utilization (177).

Disrupted peripheral clock rhythms, whether from circadian mutations or environmental perturbation (e.g., shiftwork), can lead to overweight and metabolic disease. TRF is a promising intervention in this context. Periods of fasting are likely to restore metabolic function in an otherwise obesogenic environment independent of 24-h calorie intake partly by permitting the expression of metabolic pathways inhibited by food intake. For instance, liver-specific AMPK overexpression (mimicking the fasting state) inhibited *de novo* lipogenesis and was sufficient to prevent hepatic steatosis in mice fed a high-fructose diet (178). The takeaway is therefore 2-fold: optimal nutrient intake is confined to a restricted time period during the animal's active phase and leaves a sufficiently long fasting window. A key question that remains is how long of a fasting window is necessary to see the protective benefits of TRF in humans. Is it necessary to fast as long as 18 h (108) or are 11–12 h sufficient (68)? The answer almost certainly depends on baseline metabolic health and the extent of the desired change in weight and metabolic parameters.

## Conclusion

The circadian expression of the core clock and the genes under its regulation is found not only in the master clock (i.e., SCN) but also throughout other tissues. Peripheral clocks respond not only to the synchronizing cues emanating from the light-entrained master clock but also to rhythms in feeding and fasting. Because peripheral clocks such as that of the liver are more sensitive to food as a zeitgeber, they are not necessarily coupled with the SCN when feeding and fasting rhythms are not aligned with the light/dark cycle. Furthermore, different peripheral tissues have varying degrees of responses to food intake during the inactive phase, thus potentiating peripheral misalignment. Obesogenic diets also disrupt feeding rhythms and thereby circadian metabolism. In modern humans, the discordance between behavioral and endogenous clock rhythms is prevalent, and this temporal misalignment leads to systemic metabolic dysregulation. While evening light exposure will likely continue to be a reality, food intake is a powerful zeitgeber around which behaviors are more plastic. Active phase TRF may have remarkable potential to prevent the deleterious metabolic effects of both obesogenic diets and night shift work.

## Future Directions

The relative importance of the core molecular clock as a mediator of food intake signals remains to be delineated. Does TRF affect the rhythm-generating clock itself, or does it instead affect the “hands of the clock” (outputs of the core oscillator)? The answers to these questions could be important in developing potential pharmacological interventions (e.g., fasting mimetic drugs and direct clock gene agonists) for use when lifestyle changes are not feasible, such as in the case of shift work.

Other lifestyle interventions that could influence circadian metabolism and prevent its misalignment are also being examined. Exercise in particular may influence peripheral clocks in skeletal muscles and adipose tissue (179) as it activates many of the same pathways as fasting does, and these feed back into the core clock (180). Indeed, acute exercise was found to alter the human subcutaneous adipose tissue transcriptome (181). Recently, a human phase response curve for exercise was created (182). Phase response curves illustrate the relationship between the time of zeitgeber exposure and the resultant phase shifts (advances or delays) of the clock. A phase response curve for food intake will likewise be an essential tool for understanding how to prevent or alleviate circadian misalignment by TRF.

Accessing the nutrient-responsive peripheral circadian system in humans remains challenging, and innovative methods are required to translate the large body of mechanistic work in animals. Serial sampling is necessary to observe 24-h rhythms, which presents the particular challenge for invasive studies (e.g., muscle or adipose biopsies). Nevertheless, because animal studies have shown that the circadian metabolome and its response to nutrition are highly tissue-specific (144, 145), this work is highly important. As Roenneberg and Merrow (4) indicated, in order to combat misalignment, it is necessary to understand tissue-specific variations in response to “eigen-zeitgebers” (particular/own zeitgebers, e.g., nutrients and sympathetic innervation).

Circadian transcriptomics and metabolomics studies in humans have been carried out using serial samples of plasma, muscle biopsies, and subcutaneous adipose tissue. However, further studies are needed to synthesize these large datasets toward identification of biomarkers of circadian metabolic function. Indeed, preliminary efforts are underway to identify human circadian biomarkers (183). If they are sufficiently well-defined, these biomarkers could be screened at their peaks and nadirs to determine interpretable signatures of circadian alignment and identify early markers of circadian disruption in metabolic pathophysiology. Alternatives to plasma for non-invasive serial sampling include urine (184) and breath. The latter was used in circadian metabolomics in a proof-of-principle study (185), and it has recently been highlighted as an ideal method for continuous sampling and rapid untargeted metabolomic analysis (186), making this an exciting avenue for translational work.

A key challenge in the translational application of circadian biology is the large interindividual variations in metabolite rhythms (92, 183, 187). What causes these variations, and how are they related to health outcomes? In some cases, variability may be associated with chronotype (93). The interindividual differences in the circadian metabolic response to misaligned zeitgebers, especially in the context of shift work, warrant further attention. Moreover, the response of the human circadian metabolome under various diet conditions deserves further exploration given the largescale alterations observed in mice (145). At the same time, future research in animal models may elucidate the circadian effects of specific macronutrient ratios, micronutrients and supplements [e.g., fish oil: (188)]. Together this will inform novel therapeutic approaches to combat metabolic disease in the modern environment.

## Author Contributions

LP and H-KS conceived, designed, and wrote the manuscript.

## Conflict of Interest

The authors declare that the research was conducted in the absence of any commercial or financial relationships that could be construed as a potential conflict of interest.
